# Adaptation and Validation of the Gastroesophageal Reflux Disease Questionnaire (GerdQ) for Arabic-Speaking Populations

**DOI:** 10.7759/cureus.109469

**Published:** 2026-05-22

**Authors:** Ahmad F Alenezi, Rakan M Alotaibi, Mai Helmy, Hadeel Ghazzawi, Salma Y Abu-Saleh, Khaled Trabelsi, Haitham Jahrami

**Affiliations:** 1 Department of Medicine, McGill University, Montreal, CAN; 2 Department of Medicine, Ministry of Health, Kuwait City, KWT; 3 Department of Medicine, King Faisal Specialist Hospital & Research Centre, Medina, SAU; 4 College of Education, Sultan Qaboos University, Muscat, OMN; 5 Department of Nutrition and Food Technology, University of Jordan, Amman, JOR; 6 Department Nutrition and Food Technology, Jordan University of Science and Technology, Irbid, JOR; 7 Department of Sports Medicine, University of Sfax, Sfax, TUN; 8 Department of Psychiatry, Arabian Gulf University, Manama, BHR

**Keywords:** arabic-speaking countries, arabic version, diagnosis, gastroesophageal reflux disease (gerd), gerdq, validation

## Abstract

Objective: The objective of this study was to translate, adapt, and validate the Gastroesophageal Reflux Disease Questionnaire (GerdQ) in Arabic.

Method: This study involved translating the original English version of the GerdQ into Arabic. A cross-sectional study design was employed utilizing an online Google Forms (Google LLC, Mountain View, California, United States) survey distributed across 22 Arabic-speaking countries.

Results: A total of 201 participants with a mean age of 23.94 ± 8.03 years from various Arabic countries completed the questionnaire. Of these, 145 participants were female (72%). The Arabic version of the GerdQ demonstrated excellent reliability, with an internal consistency of 0.86. The test-retest reliability of the questionnaire over the two-week interval was 0.92. The Comparative Fit Index (0.96), Tucker-Lewis index (0.94), and other incremental fit indices were all above the 0.95 threshold, which is indicative of good fit. The questionnaire demonstrated moderate diagnostic performance for detecting GERD (area under the curve (AUC) = 0.67). An optimal cutoff score of 10 maximized the sensitivity, specificity, and other metrics.

Conclusion: The newly adapted Arabic version of the GerdQ is anticipated to enhance the effectiveness of this tool in both research and clinical settings within Arabic-speaking regions, facilitating better understanding and management of gastroesophageal reflux disease.

## Introduction

Gastroesophageal reflux disease (GERD) is defined as a condition characterized by chronic backflow of gastric contents from the stomach to the esophagus, oral cavity, or lungs [1]. The typical GERD symptoms include heartburn and acid reflux. There are distinct histological changes in GERD, which include nonerosive reflux disease (NERD) and Barrett's esophagus [2]. GERD also causes extraesophageal symptoms, such as sore throat, throat clearing, asthma, cough, and unexplained chest pain [3]. Its complications include stricture, ulceration, metaplasia, and esophageal adenocarcinoma [4]. It has a major impact on quality of life due to its chronic nature. Nonerosive reflux disease is associated with abnormal exposure to esophageal acid without endoscopic mucosal injuries, and erosive esophagitis is characterized by mucosal breaks observed via endoscopy [3,5-7].

The natural progression of GERD is incompletely understood; however, evidence suggests that there is limited progression (from GERD to erosive esophagitis and from erosive esophagitis to Barrett's esophagus) and regression (from erosive esophagitis to nonerosive reflux disease) over time [8-10]. Given its high prevalence and chronicity, GERD imposes a significant economic burden on affected individuals and health services [11]. GERD is one of the most commonly diagnosed digestive disorders in the United States, with an approximate prevalence of 20%, resulting in a significant financial burden and adverse quality of life [12,13].

The diagnosis of GERD is typically established through a combination of clinical features, improvement with acid suppressive therapies, and, in some cases, objective testing via upper endoscopy and esophageal pH monitoring. Common questionnaires used to aid in the diagnosis of GERD include the Reflux Disease Questionnaire (RDQ) [14], Carlsson-Dent questionnaire [15], GERD Impact Scale (GIS) [16], and Gastroesophageal Reflux Disease Questionnaire (GerdQ) [17].

The GerdQ is one of the most commonly used questionnaires for diagnosing GERD. It is a six-item, easy-to-administer questionnaire that was developed as a diagnostic tool for GERD in primary care settings, and a score of 8 or higher suggests an increased likelihood of GERD [17]. The diagnostic accuracy of the GerdQ was comparable to that of a standard symptom-based diagnosis conducted by a gastroenterologist, and the GerdQ exhibited slight improvement over the symptom-based diagnosis of GERD in primary care.

In terms of the prevalence of GERD in Arabic-speaking countries, multiple studies have been performed using a self-translated Arabic version of the GerdQ. One study employing the GerdQ questionnaire reported a prevalence of 20.6% among a total of 1517 participants in the eastern region of Saudi Arabia [18]. Another study utilized the same questionnaire in Riyadh, Saudi Arabia, and included 1256 participants, and the overall prevalence in the surveyed population was 45.4% [19]. Additionally, in Egypt, a cross-sectional study using a translated Arabic version of the GerdQ was carried out among 964 medical students across six different universities and showed that 17.1% of the participants reported GERD symptoms [20]. However, these studies utilized an unvalidated translation of the Arabic version. It is important to note that the Arabic language is complex and has many dialects. The absence of a validated standardized Arabic version of the GerdQ poses a substantial risk to epidemiological studies and clinical practices. Reliance on unvalidated translations may introduce cultural and linguistic biases, leading to an inaccurate representation of the true prevalence of GERD. This, in turn, may result in suboptimal treatment strategies and an underestimation of the burden of GERD in affected populations, which include 22 Arabic-speaking countries.

This study, thus, aimed to establish a reliable and validated Arabic version of the GerdQ to ensure diagnostic accuracy similar to that of the original GerdQ.

## Materials and methods

This was a cross-sectional study conducted across 22 Arabic-speaking countries between September 28 and December 12, 2023. The survey was created using Google Forms (Google LLC, Mountain View, California, United States). Initially, a pilot study was conducted using a sample of 20 people to ensure that the questionnaire was understandable and clear. Approval for the use, translation, and validation of the GERD questionnaire was obtained from Prof. Roger Jones, the developer of the GerdQ. Additionally, ethical approval was secured from the Research Committee of Menoufia University (approval letter ID: 12142023). All the methods used in this study adhered to the ethical guidelines of the 1964 Helsinki declaration and its subsequent modifications. Informed consent was obtained from all participants.

Study population

Participants were recruited via online distribution of the survey through social media platforms (WhatsApp (Meta Platforms, Inc., Menlo Park, California, United States), X (X Corp., Bastrop, Texas, United States), and Instagram (Meta Platforms, Inc.)). All respondents diagnosed with GERD at primary care or hospital settings above the age of 18 were included. All respondents who self-diagnosed and treated with GERD without formal physician assessment were excluded from the study. A total of 201 participants were finally included.

Translation methods

A back translation method was used [21]. Initially, two bilingual experts who were native Arabic speakers translated the GerdQ from English to Arabic. Changes were made to the grammar, and adaptations were made to align with the Arabic linguistic, social, and cultural contexts while ensuring that the semantic context remained unchanged. After this, the translated Arabic draft was sent to two bilingual translators separately to translate the Arabic draft back to English; they did not read the original English version of the scale. The Arabic version and its back-translated English counterpart underwent a process of evaluation, comparison, and matching. The entire process was carried out under the supervision of an external certified language translator. The research team finalized the Arabic version of the GerdQ questionnaire after receiving feedback from a certified language translator. Table 1 presents a comparison of the original GerdQ scale items with their corresponding translations in Arabic.

**Table 1:** Comparison of the original GerdQ scale items with their corresponding Arabic translations. GerdQ: Gastroesophageal Reflux Disease Questionnaire Approval for the use, translation, and validation of the GERD questionnaire was obtained from Prof. Roger Jones, the developer of the GerdQ [17].

Item	Original GerdQ	Arabic Version GerdQ
1	How often did you have a burning feeling behind your breastbone (heartburn)	كم مرة شعرت بالحرقة خلف عظمة الصدر (حرقة المعدة)؟
2	How often did you have stomach contents (liquid or food) moving upward to your throat or mouth (regurgitation)	كم مرة تحركت لديك محتويات المعدة (السوائل او الطعام) الى اعلى الحلق او الفم (الارتجاع)؟
3	How often did you have a pain in the center of the upper stomach?	كم مرة شعرت بألم في وسط الجزء العلوي من المعدة (رأس المعدة)؟
4	How often did you have nausea	كم مرة شعرت بالغثيان (اللوعة)؟
5	How often did you have difficulty getting a good night’s sleep because of your heartburn and⁄or regurgitation?	كم مرة واجهتك صعوبة في الحصول على نوم جيد أثناء الليل النوم بسب حرقة المعدة و/أو الارتجاع؟
6	How often did you take additional medication for your heartburn and⁄or regurgitation, other than what the physician told you to take? (such as Tums, Rolaids, Maalox?)	كم مرة تناولت دواء إضافي لحرقة المعدة و/أو الارتجاع غير الذي اخبرك الطبيب بتناوله؟ (مثل شراب او حبوب مضادة الحموضة، جافيسكون او مالوكس او غيرها)

Data collection process and statistical analysis

Descriptive statistics were calculated to summarize the sample and scale characteristics. Reliability and validation analyses were subsequently conducted to evaluate the psychometric properties of the GerdQ for assessing GERD symptom severity. Internal reliability was examined using McDonald’s ω [22], Cronbach’s α [23], and Guttman’s λ [24] coefficients. Confirmatory factor analysis was performed to test the unidimensional scale structure, with all 6 items loading on a single GERD symptom factor [25]. Maximum likelihood estimation was used to determine the model parameters, and fit was assessed using the comparative fit index (CFI), Tucker-Lewis index (TLI), root mean square error of approximation (RMSEA), standardized root mean square residual (SRMR), and other absolute and incremental indices. Values above 0.9 for the CFI and TLI were considered indicators of good fit. RMSEA and SRMR values less than 0.08 and 0.05, respectively, were used as cutoffs for adequate model fit, as suggested by Hu and Bentler [26].

Reliability coefficients above 0.7 and significant factor loadings provided quantitative evidence for scale validation. These analyses evaluated the reliability of the GerdQ scores and the validity of the questionnaire for assessing the underlying construct of reflux-related symptom severity in this population. To evaluate the test-retest reliability of the questionnaire, a random subsample of 100 participants completed the questionnaire a second time approximately two weeks after their initial completion. Administering the questionnaire to the same respondents twice allowed us to assess the stability and consistency of the questionnaire by analyzing the correlation between individual respondents' scores at two different time points. A high correlation between the Time 1 and Time 2 scores suggested good test-retest reliability, indicating that the questionnaire consistently measures the constructs it is intended to measure. Examining test-retest reliability helps ensure that the questionnaire produces stable, replicable results over time. Cohen's kappa coefficient was used to assess the test-retest reliability of the questionnaire [27].

The diagnostic accuracy of the questionnaire for GerdQ was evaluated by calculating the sensitivity, specificity, positive predictive value (PPV), negative predictive value (NPV), Youden's index, area under the receiver operating characteristic curve (AUC), and overall metric score at each possible cutoff point from 3 to 15. Sensitivity was defined as the proportion of true positives correctly identified by the questionnaire, while specificity was the proportion of true negatives correctly identified. PPV and NPV represented the probabilities of having or not having disease X given a positive or negative questionnaire result, respectively. Youden's index assessed overall diagnostic effectiveness as sensitivity + specificity - 1. The AUC was used to measure overall diagnostic discrimination, with higher values indicating better accuracy. The metric score (sensitivity + specificity - AUC) provided a composite indicator of the questionnaire's performance at each cutoff point, with higher scores indicating better results [28].

All analyses were performed using R statistical software version 4.2.3 "Shortstop Beagle" (Released March 15, 2023; R Foundation for Statistical Computing, Vienna, Austria, https://cran.r-project.org/bin/windows/base/old/4.2.3/), with a P-value of 0.05 used to determine statistical significance.

## Results

Our cross-sectional study surveyed 201 participants to evaluate symptoms of GERD using an Arabic version of GerdQ. The mean age of the participants was 23.94 years (SD = 8.03), and most participants were female (n=145, 72%) and university students (n=185, 92%) (Table 2).

**Table 2:** Demographical and clinical characteristics of participants (N=201) Data presented as frequency (percentage), except for age, which is presented as mean±SD

Characteristics		Values
Age		23.94±8.03
Sex	Female	145 (72%)
	Male	56 (28%)
University Student		185 (92%)
Formally diagnosed gastroesophageal reflux disease		28 (14%)
Symptoms for more than a year		69 (34%)
Underwent esophageal acidity monitoring device (pH monitoring)		3 (1%)
Performed upper gastrointestinal endoscopy (gastroscopy)		11 (5%)

The total mean GerdQ symptom score was 8.96 (SD = 2.01) out of a maximum score of 18 (Table 3). The highest score for individual symptoms on the GerdQ was obtained for GerdQ item 3, related to sleep disturbances due to reflux (mean 3.13, SD = 0.97), while the lowest score was obtained for GerdQ item 6, related to the impact of symptoms on work/daily life (mean 0.39, SD = 0.79). Only 28 participants (14%) had a formal diagnosis of GERD, although 69 (34%) reported experiencing symptoms for more than a year. The rates of prior esophageal pH monitoring (n=3, 1%) and endoscopy (n=11, 5%) were very low, suggesting a disconnect between symptom burden and formal diagnostic testing.

**Table 3:** Mean score in each item of the Arabic GerdQ GerdQ: Gastroesophageal Reflux Disease Questionnaire

GerdQ Items	Mean	SD
Item 1	0.79	0.93
Item 2	0.93	0.96
Item 3	3.13	0.97
Item 4	3	0.99
Item 5	0.71	0.94
Item 6	0.39	0.79
GERDQ Total	8.96	2.01

Reliability analysis revealed that the GerdQ has high internal consistency for measuring GERD symptoms in this sample. McDonald's ω, Cronbach's α, and Guttman's λ6 estimates were all 0.86, indicating excellent composite reliability. Detailed results are presented in Table 4. The confidence intervals (CIs) around these point estimates ranged from 0.81 to 0.89. The item-total correlations were acceptable, ranging from 0.43 to 0.76. Removal of any individual item resulted in slightly lower reliability coefficients overall between 0.83 and 0.85. The test-retest reliability of the questionnaire over the two-week interval was 0.94.

**Table 4:** Confirmatory factor analysis of the GerdQ (N = 201) GerdQ: Gastroesophageal Reflux Disease Questionnaire

Factor	Indicator	Estimate	Std. Error	z value	p
Gastroesophageal Reflux Disease	GerdQ item 1	0.71	0.06	11.98	<0.001
	GerdQ item 2	0.72	0.06	11.67	<0.001
	GerdQ item 3	0.69	0.06	10.99	<0.001
	GerdQ item 4	0.64	0.07	9.53	<0.001
	GerdQ item 5	0.71	0.06	11.71	<0.001
	GerdQ item 6	0.51	0.05	9.71	<0.001

Confirmatory factor analysis (CFA) provides evidence validating the unidimensional factor structure of the GerdQ for assessing GERD symptom severity. All six GerdQ items loaded significantly onto a single factor, representing overall GERD symptomatology, with factor loadings ranging from 0.51 to 0.72 (all P-value < 0.001). The items with the strongest associations with the underlying GERD factor were GerdQ item 2 (How often did you have stomach contents (liquid or food) moving upward to your throat or mouth (regurgitation); factor loading = 0.72) and GerdQ item 5 (How often did you have difficulty getting a good night’s sleep because of your heartburn and/or regurgitation; factor loading = 0.71). The item with the lowest, but still significant, factor loading was GerdQ item 6 (impact on work/daily life; factor loading = 0.51). Results are shown in Table 5.

**Table 5:** Reliability analysis coefficients of the GerdQ (N = 201) GerdQ: Gastroesophageal Reflux Disease Questionnaire

Estimate	McDonald's ω	Cronbach's α	Guttman's λ6
Point estimate	0.86	0.86	0.85
95% CI	0.83-0.89	0.83-0.89	0.81-0.88
If item dropped			
GerdQ item 1	0.83	0.83	0.81
GerdQ item 2	0.83	0.83	0.80
GerdQ item 3	0.84	0.84	0.81
GerdQ item 4	0.85	0.85	0.82
GerdQ item 5	0.83	0.83	0.80
GerdQ item 6	0.85	0.85	0.83

The GerdQ demonstrated good model fit across several absolute and incremental indices, which are presented in Table 6. The comparative fit index (CFI=0.96), Tucker-Lewis index (TLI=0.94), and other incremental fit indices were all above the 0.95 threshold, which is indicative of good fit. High-norm (0.91-0.95) and non-norm (0.94-0.96) incremental indices also provide support that the single-factor model better fits the data than does the null model. For the absolute fit, the standardized root mean square residual was low at 0.03, and the goodness-of-fit index was 1. The root mean square error of approximation was 0.1, with the 90% CI ranging from 0.06 to 0.14, hovering around the more liberal cutoff of less than 0.1 for an acceptable fit. Information criteria such as Akaike’s and Bayesian estimators also indicated strong parsimonious fits. At the same time, the RMSEA P-value (0.03) suggested a marginal fit; the preponderance of indicators within recommended ranges provided quantitative validation of the unidimensional factor structure capturing common GERD symptom variance.

**Table 6:** Fitness indices of the GerdQ (N = 201) GerdQ: Gastroesophageal Reflux Disease Questionnaire

Metric	Value
Comparative Fit Index (CFI)	0.96
Tucker‒Lewis Index (TLI)	0.94
Parsimony Normed Fit Index (PNFI)	0.57
Bollen's Relative Fit Index (RFI)	0.91
Bollen's Incremental Fit Index (IFI)	0.96
Relative Noncentrality Index (RNI)	0.96
Log-likelihood	-1386.05
Number of free parameters	18
Akaike (AIC)	2808.09
Bayesian (BIC)	2867.55
Sample-size adjusted Bayesian (SSABIC)	2810.53
Root mean square error of approximation (RMSEA)	0.1
RMSEA 90% CI lower bound	0.06
RMSEA 90% CI upper bound	0.14
RMSEA P-value	0.03
Standardized root mean square residual (SRMR)	0.03
Hoelter's critical N (α = .05)	128.16
Hoelter's critical N (α = .01)	163.83
Goodness-of-fit index (GFI)	1
McDonald fit index (MFI)	0.96
Expected cross validation index (ECVI)	0.31

A cutoff value of 8 demonstrated high sensitivity coupled with low specificity, quantified at 85.71% and 18.5%, respectively. Conversely, adopting a threshold value of 9 resulted in a sensitivity of 64.29% and a specificity of 54.34%. Notably, a more harmonious balance between sensitivity and specificity was observed when the cutoff point was set at 10, for which the sensitivity and specificity were 64.29% and 71.1%, respectively.

## Discussion

This study aimed to establish a reliable and validated Arabic version of the GerdQ, addressing a crucial gap in the assessment and epidemiological understanding of GERD in Arabic-speaking populations by adapting the scale to a clear standard Arabic language to be used across 22 Arabic-speaking countries.

In the original GerdQ development study [17], which used data from the DIAMOND study [29], a cutoff point of 8 was proposed for testing for GERD, with sensitivity and specificity of 64.6% and 71.4%, respectively. Subsequently, an alternative validation study identified a cutoff point of 9 as more effective, yielding improved sensitivity and specificity of 66% and 64%, respectively [30]. A Japanese validation study conducted in general and hospital-based populations found that a GerdQ cutoff score of 8 was appropriate for the Japanese population; in the hospital-based cohort, this cutoff predicted reflux esophagitis with a sensitivity of 34.3% and specificity of 82.5% [31]. In our validation study, a cutoff value of 10 demonstrated a sensitivity of 64.29% and specificity of 71.1%, which were comparable to those observed in the original GerdQ study. Table 7 presents GerdQ performance metrics at various cutoff points.

**Table 7:** Performance metrics of the GerdQ at various cutoff points. PPV: positive predictive value; NPV: negative predictive value; AUC: area under the receiver operating characteristic curve; GerdQ: Gastroesophageal Reflux Disease Questionnaire

Cutpoint	Sensitivity (%)	Specificity (%)	PPV (%)	NPV (%)	Youden's Index	AUC	Metric Score
3	100%	0%	13.93%	NaN%	0.00	0.67	1.00
4	96.43%	0%	13.5%	0%	-0.04	0.67	0.96
5	96.43%	0.58%	13.57%	50%	-0.03	0.67	0.97
6	92.86%	1.73%	13.27%	60%	-0.05	0.67	0.95
7	92.86%	5.2%	13.68%	81.82%	-0.02	0.67	0.98
8	85.71%	18.5%	14.55%	88.89%	0.04	0.67	1.04
9	64.29%	54.34%	18.56%	90.38%	0.19	0.67	1.19
10	64.29%	71.1%	26.47%	92.48%	0.35	0.67	1.35
11	46.43%	86.13%	35.14%	90.85%	0.33	0.67	1.33
12	32.14%	92.49%	40.91%	89.39%	0.25	0.67	1.25
13	25%	94.8%	43.75%	88.65%	0.20	0.67	1.20
14	17.86%	99.42%	83.33%	88.21%	0.17	0.67	1.17
15	7.14%	100%	100%	86.93%	0.07	0.67	1.07

Strengths

The strength of the validated Arabic version of the GerdQ is evident in three main points. First, the reliability analysis demonstrated high internal consistency, with multiple coefficients (McDonald's ω, Cronbach's α, and Guttman's λ6) consistent at approximately 0.86. This level of reliability is crucial for a diagnostic tool, ensuring that the scale consistently measures what it is intended to do.

Second, the confirmatory factor analysis further strengthens the validity of the GerdQ. The significant estimates and low P-values for the indicators suggest that the items on the GerdQ scale are effective at measuring GERD. These results are critical for ensuring the scale's construct validity, indicating that the scale accurately measures the GERD construct as theorized.

Finally, the model fit indices indicate an excellent fit of the confirmatory factor analysis model with the observed data. Indices such as the CFI and TLI, which are above 0.90, suggest that the model accurately represents the data, further validating the Arabic version of the GerdQ.

Additionally, the scale's design does not benefit from the elimination of any factor, underscoring its comprehensive and cohesive nature. The collective contribution of each item on the scale to its overall high reliability and strong construct validity further reinforces the effectiveness and appropriateness of the GerdQ in these settings. This thorough validation process established the Arabic version of the GerdQ as a robust and well-structured tool for the accurate assessment of GERD symptoms, making it a reliable resource in both clinical and research domains.

Limitations

While our study provides valuable insights, it is not without limitations. The use of an online survey and the predominant participation of university students might limit the generalizability of our findings. Second, the adaptation of the GerdQ into classical Arabic, which is distinct from the colloquial or standard dialects, requires certain considerations. This choice of language, while ensuring a standardized form, may not be universally comprehensible across all Arabic-speaking regions, particularly in North African countries where local dialects and linguistic variations are prevalent. Nonetheless, individuals familiar with classical Arabic are expected to be able to comprehend the questionnaire effectively.

## Conclusions

The Arabic version of the GerdQ was validated thoroughly and demonstrated robust reliability and validity. The meticulous adaptation and validation process ensured that the tool was culturally and linguistically appropriate for Arabic-speaking populations, thereby enhancing its applicability in diverse settings. Overall, the successful adaptation and validation of the Arabic version of the GerdQ is anticipated to enhance the effectiveness of this tool in both research and clinical settings within Arabic-speaking regions, facilitating better understanding and management of GERD.
